# Swine influenza viruses isolated in 1983, 2002 and 2009 in Sweden exemplify different lineages

**DOI:** 10.1186/1751-0147-52-65

**Published:** 2010-12-14

**Authors:** István Kiss, Ádám Bálint, Giorgi Metreveli, Eva Emmoth, Frederik Widén, Sándor Belák, Per Wallgren

**Affiliations:** 1 National Veterinary Institute, Ulls väg 2B, 75189 Uppsala, Sweden; 2 Department of Virology, Central Agricultural Office, Veterinary Diagnostic Directorate, Tábornok u. 2., H-1149 Budapest, Hungary; 3 Department of Microbiology, Central Agricultural Office, Veterinary Diagnostic Directorate, Bornemissza u. 3-7, H-4031 Debrecen, Hungary; 4 Department of Biomedical Sciences and Veterinary Public Health, Division of Microbiology and Food Safety, Swedish University of Agricultural Sciences, Box 7036, S-75007 Uppsala, Sweden; 5 Department of Clinical Sciences, Swedish University of Agricultural Sciences, Box 7054, S-75007 Uppsala, Sweden

## Abstract

Swine influenza virus isolates originating from outbreaks in Sweden from 1983, 2002 and 2009 were subjected to nucleotide sequencing and phylogenetic analysis. The aim of the studies was to obtain an overview on their potential relatedness as well as to provide data for broader scale studies on swine influenza epidemiology. Nonetheless, analyzing archive isolates is justified by the efforts directed to the comprehension of the appearance of pandemic H1N1 influenza virus. Interestingly, this study illustrates the evolution of swine influenza viruses in Europe, because the earliest isolate belonged to 'classical' swine H1N1, the subsequent ones to Eurasian 'avian-like' swine H1N1 and reassortant 'avian-like' swine H1N2 lineages, respectively. The latter two showed close genetic relatedness regarding their PB2, HA, NP, and NS genes, suggesting common ancestry. The study substantiates the importance of molecular surveillance for swine influenza viruses.

## Findings

The records of the swine influenza history in Europe start with H1N1 viruses dating back to 1938, when an H1N1 strain of early human influenza virus gene pool was isolated from pigs in Great Britain [1]. In 1950, classical swine influenza viruses (SIVs) were isolated in Czechoslovakia but after that no swine influenza virus detection was reported for more than two decades [1,2]. Between the mid 1970 s and mid 1980 s, human origin H3N2 influenza viruses and classical SIVs circulated simultaneously. In 1979, an H1N1 influenza virus of avian origin was first detected in pigs, in Belgium, which gradually replaced the classical viruses and became dominant in Europe [3,4]. Reassortment events between human origin H3N2 and avian-like H1N1 SIVs resulted in the emergence of H3N2 viruses possessing surface glycoprotein genes of human and internal genes of avian origin. These viruses eventually superseded the former H3N2 viruses of human origin around 1983-84 [5,6], but their current presence show great variation across Europe [1]. As a results of further reassortment events several kinds of H1N2 subtype SIVs arose: the first case was reported in France in 1987 [7]. This virus comprised HA of avian and NA of human origin, which had not become widespread among the European pig population. In 1994, a further reassortant variant was identified in the UK, comprising haemagglutinin (HA) and neuraminidase (NA) genes of human origin and internal genes of avian origin ('human-like' reassortant SIV H1N2 viruses)[8]. The different H1N2 viruses have reassorted with each other as well as with avian-like H1N1 viruses resulting in difficulties to characterize and classify them [1]. Recently, novel reassortant H1N2 SIVs were isolated in Germany and in Italy, having a mixture of the characteristics of porcine H1N2 and H3N2 viruses [1,9]. In 2006, a further type of reassortant was isolated in Italy, belonging to H3N1 subtype SIVs, which plausibly arose by the exchange of genetic material between H1N1 and H3N2 SIVs [10]. The occurrence and prevalence of SIVs vary among the different regions in Europe but the avian origin H1N1 appears to be the most predominant subtype, followed by reassortant H3N2 and to a lower extent by the H1N2 subtypes, which comprise viruses of diverse genetic constellation [1,11].

Our goal was to investigate the genetic composition of the available archive and recent Swedish SIV isolates in order to establish their relation to the existing lineages and to find out if they were genetically or epidemiologically related to each other, and further, to provide data for broader scale studies on swine influenza virus ecology and evolution. The viruses were collected in 1983, 2002 and 2009, from swine influenza outbreaks in Sweden [12,13]. Prior to the 1983 outbreak there was no evidence of the presence of swine influenza in Sweden [12]. The isolates were subjected to PCR and nucleotide sequencing to obtain representative portions of the viral genome by using the method described and applied earlier [13,14]. The genetic material of A/swine/Lidköping/1193/02(H1N1) virus was amplified directly from nasal swabs collected and stored from a pig herd showing respiratory clinical signs, which subsequently was diagnosed as swine influenza. The obtained samples were processed as mentioned above. For the phylogenetic analyses closely related nucleotide sequences were collected based on the BLAST search, while additional sequences, considering the literature, were obtained from the Influenza Virus Resource platform of NCBI http://www.ncbi.nlm.nih.gov/genomes/FLU/FLU.html. The genes were assigned to lineages according to the Influenza A Virus Genotype Tool [15] and in the case of HA and NA also in accordance with Liu et al. [16], who proposed a comprehensive nomenclature system for influenza A viruses based on the panorama phylogeny of approximately 23 000 sequences related to the surface glycoproteins. Sequence assembly, multiple alignment and alignment trimming were performed with the CLC Main Workbench 5.5 (CLC bio A/S, Aarhus, Denmark). Distance based neighbor-joining and character based maximum parsimony phylogenetic trees were generated using the Molecular Evolutionary Genetics Analysis (MEGA) software v.4.0. [17]. The topology of the trees was confirmed with 1000 bootstrap replicates. For the neighbor-joining trees, the Kimura 2-parameter method was used [18]. Other models tested showed similar results. The nucleotide sequences obtained by this study were deposited in the GenBank (accession numbers GU236506-GU236521).

**Figure 1:**
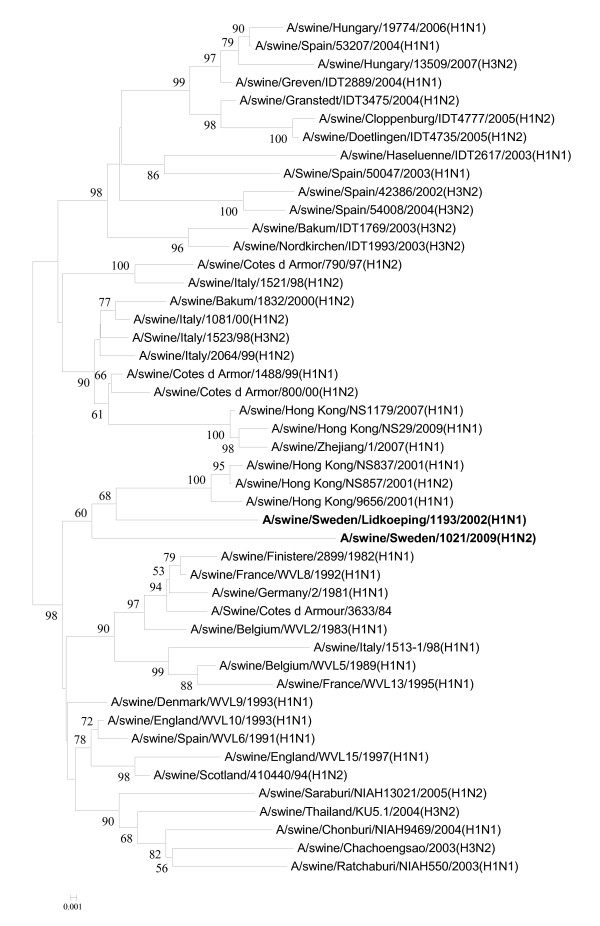
**Evolutionary relationships of the HA genes of A/swine/Skåne/1321/1983(H1N1), A/swine/Lidköping/1193/02 (H1N1) and A/swine/Sweden/1021/09 (H1N2)**. The phylogenetic tree was generated by the neighbor-joining method. Bootstrap values of 1000 resamplings in per cent are indicated at key nodes. Bootstrap values above 50% are shown. The Swedish viruses are highlighted with bold letters and the lineages are indicated according to the Influenza A Virus Genotype Tool [15].

**Figure 2:**
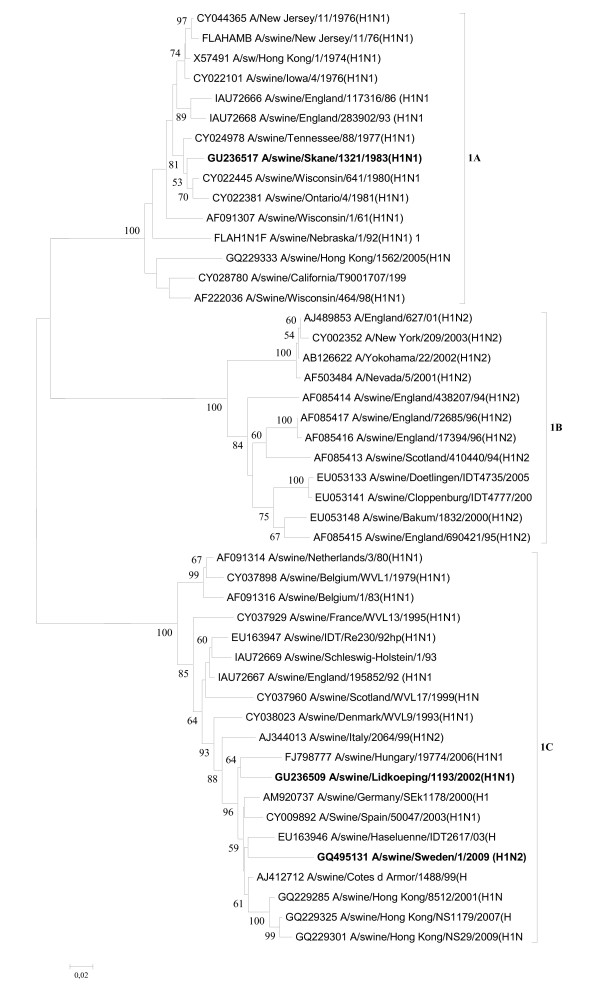
**Evolutionary relationships of the NA genes of A/swine/Skåne/1321/1983(H1N1), A/swine/Lidköping/1193/02 (H1N1) and A/swine/Sweden/1021/09 (H1N2)**. The phylogenetic tree was generated by the neighbor-joining method. Bootstrap values of 1000 resamplings in per cent are indicated at key nodes. Bootstrap values above 50% are shown. The Swedish viruses are highlighted with bold letters and the lineages are indicated according to the Influenza A Virus Genotype Tool [15].

The isolates were genotyped as follows: A/swine/Skåne/1321/1983(H1N1) [B, A, C, 1A, A, 1B,A, 1A], A/swine/Lidköping/1193/02(H1N1) [F, G, I, 1C, F, 1F, F, 1E], and A/swine/Sweden/1021/09(H1N2) [F, G, I, 1C, F, 2A, F, 1E], indicating that the two more recent isolates showed more nucleotide homology with each other than with the one from 1983. Abusugra et al. [12,19] found that isolate A/swine/Skåne/1321/83(H1N1) was very similar to that time "US viruses", now termed classical SIVs, by using haemagglutination and neuraminidase inhibition, as well as oligonucleotide fingerprinting methods. Phylogenetic analysis of the partial nucleotide sequences of each viral gene confirmed the previous findings (for HA and NA see Figures 1 and 2).

**Figure 3:**
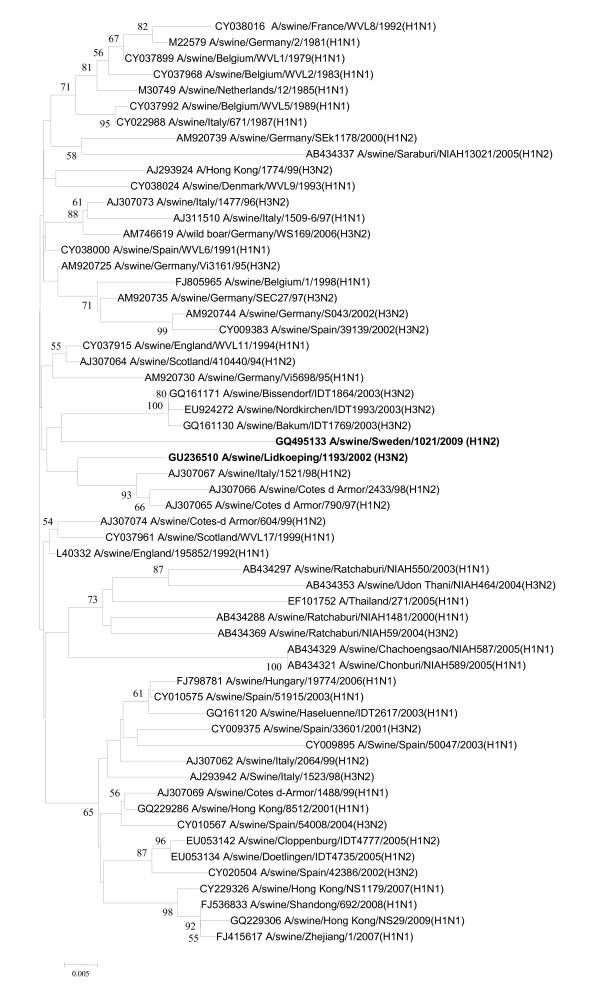
**Evolutionary relationships of the PB2 genes of A/swine/Skåne/1321/1983(H1N1), A/swine/Lidköping/1193/02 (H1N1) and A/swine/Sweden/1021/09 (H1N2)**. The phylogenetic tree was generated by the neighbor-joining method. Bootstrap values of 1000 resamplings in per cent are indicated at key nodes. Bootstrap values above 50% are shown. The Swedish viruses are highlighted with bold letters and the lineages are indicated according to the Influenza A Virus Genotype Tool [15].

**Figure 4:**
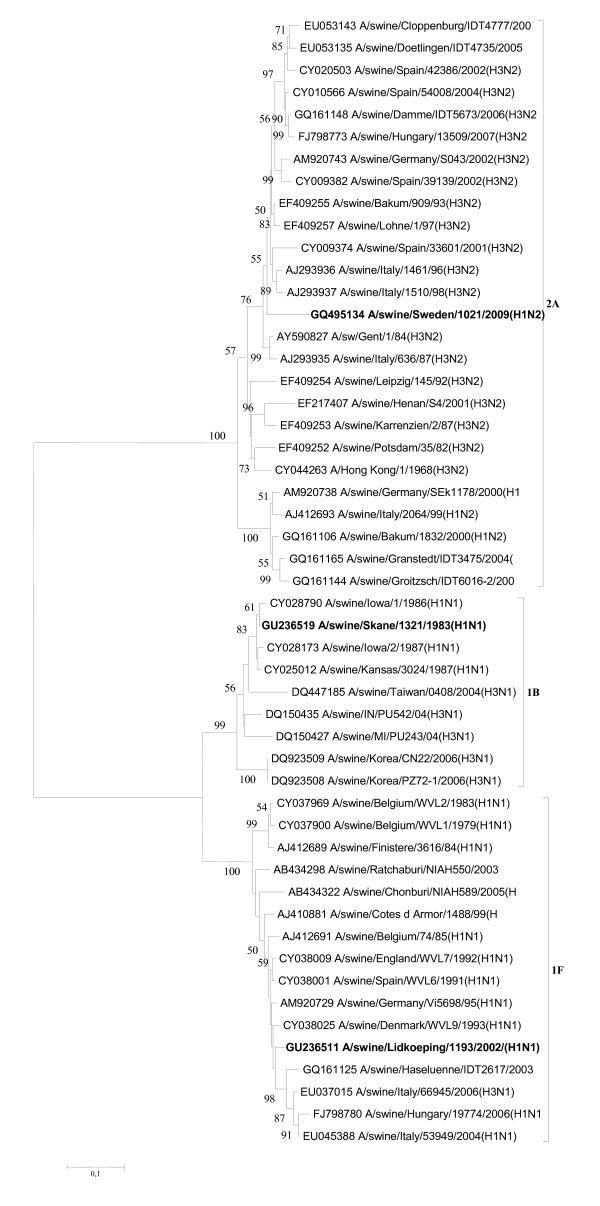
**Evolutionary relationships of the NP genes of A/swine/Skåne/1321/1983(H1N1), A/swine/Lidköping/1193/02 (H1N1) and A/swine/Sweden/1021/09 (H1N2)**. The phylogenetic tree was generated by the neighbor-joining method. Bootstrap values of 1000 resamplings in per cent are indicated at key nodes. Bootstrap values above 50% are shown. The Swedish viruses are highlighted with bold letters and the lineages are indicated according to the Influenza A Virus Genotype Tool [15].

**Figure 5:**
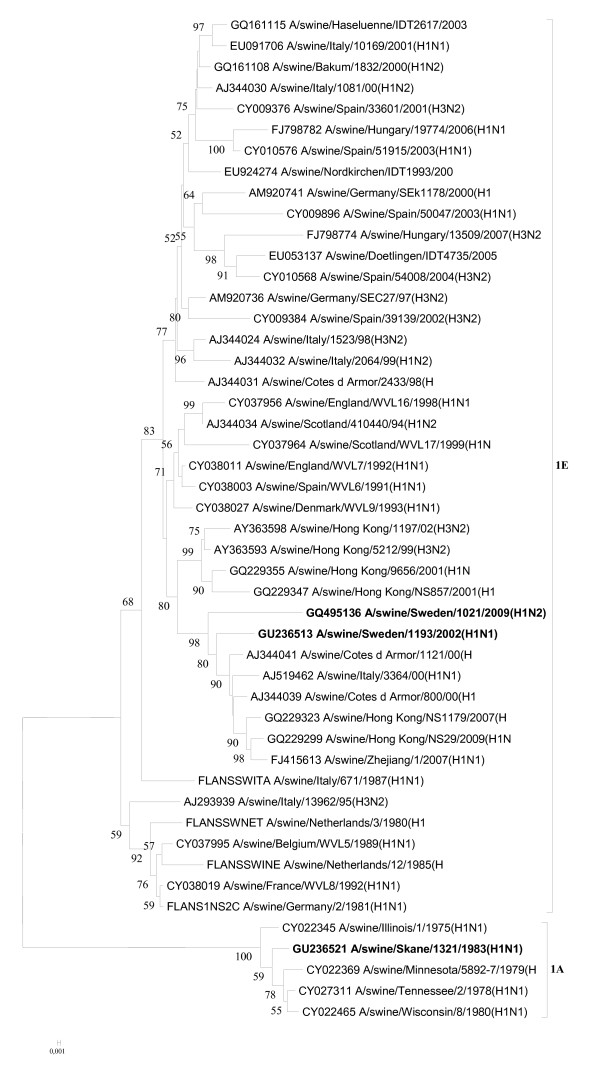
**Evolutionary relationships of the NS genes of A/swine/Skåne/1321/1983(H1N1), A/swine/Lidköping/1193/02 (H1N1) and A/swine/Sweden/1021/09 (H1N2)**. The phylogenetic tree was generated by the neighbor-joining method. Bootstrap values of 1000 resamplings in per cent are indicated at key nodes. Bootstrap values above 50% are shown. The Swedish viruses are highlighted with bold letters and the lineages are indicated according to the Influenza A Virus Genotype Tool [15].

Both A/swine/Lidköping/1193/02(H1N1) and A/swine/Sweden/1021/09(H1N2) proved to be European SIV variants, the latter one belonging to the 'avian-like' (so-called 'second-generation') reassortant H1N2 viruses, similar to those earlier detected in France, Italy and recently in Denmark [1,13,20]. Sequence comparison of the relevant genes of the isolates from 2002 and 2009 showed the following nucleotide identities: PB2 (93.45%), PB1 (90.99%), PA (90.74%), HA (92.59%), NP (94.90%), M1 (95.41%) and NS1 (94.64%). Regarding the internal genes the phylogenetic analyses revealed that they had closely related PB2, NP and NS genes, and grouped together with SIVs isolated in Hong Kong (i.e., A/swine/Hong Kong/NS857/2001(H1N2)) at the beginning of the last decade [21] (Figures 3, 4, and 5).

The matrix genes of the Swedish isolates did not show close phylogenetic associations (data not shown): A/swine/Lidköping/1193/02(H1N1) grouped together with SIVs isolated in Thailand in 2005, which obtained its M from Eurasian strains [22], while A/swine/Sweden/1021/09(H1N2) was related to German strains in this genome segment (e.g., A/swine/Muesleringen-S./IDT4263/05(H3N2). In contrast, the NS genes of the two more recent Swedish strains were closely related to each other (belonging to 1E lineage), and particularly to H1N1 and H1N2 viruses of French and Asian origin, while the 1983 isolate had an 1A lineage NS gene, a characteristic of classical SIVs (Figure 5).

The HA genes of the two more recent Swedish isolates (i.e., from 2002 and 2009) grouped together with other European lineage 1C HA genes (h1.1.3 sublineage by Liu et al., [16]), which is a descendant of an avian virus lineage (Figure 1) [3]. The 1983 isolate had a lineage 1A (h1.3.2 sublineage, Liu et al., [16]) HA, which is associated with classical SIV isolates.

The NA genes represented three lineages: 1B, 1F, and 2A corresponding to the 1983, 2002, and 2009 viruses, respectively, according to the Influenza A Virus Genotype Tool [15]. 1B (sublineage n1.3.2 in accordance with Liu et al., [16]) NAs are the characteristics of classical SIVs. NA 1F (n1.1.7) is represented by A/swine/Belgium/WVL1/1979(H1N1), which was the first SIV isolate of H1N1 avian influenza virus origin in Europe [21], and NA 2A (n2.2.3) belongs to a large number of influenza viruses of diverse origin, comprising avian, swine, and mainly human strains, including the A/Hong Kong/1/1968(H3N2) pandemic isolate [23]. The phylogenetic position of the NA gene of the 2002 isolate was alike its HA while the 2009 virus had its NA gene originated from European SIV-like H3N2 viruses (Figure 2) [13].

Taken together, the nucleotide sequence analysis confirmed that the 1983 outbreak was caused by a classical SIV. After some reports about its presence in Europe in the first half of the last century, this type of SIVs was reintroduced into Europe and isolated first in Italy in the late 70'ies supposedly by imported pigs from the US [24]. The following two outbreaks in Sweden were caused by European avian-like SIVs, the most recent one of them being a 'second generation' H1N2 reassortant, which were occasionally isolated in France and Italy in the last decade and in Denmark lately [1,13,25]. Due to the lack of nucleotide sequence information of the Danish viruses their genetic relationship to the Swedish H1N2 SIVs could not be investigated. Nevertheless, the PB2, HA, NP and NS genes of the two latter Swedish SIVs appeared to have a relative recent common ancestor. Along with that, at PB2 627 position, which is considered to play important role in the virulence of influenza strains [26], the 2002 H1N1 and the 2009 H1N2 viruses contained glutamic acid, as most avian isolates [27], while the "classic" Swedish virus a lysine. Similarly, and in accordance with the prevalent European SIVs, the two recent strains possessed the M2 S31N substitution, indicative of amantadine resistance [28], while this is missing from the classical isolate. The phylogenetic position of the isolates and the length of the respective branches did not indicate a direct parent-progeny relationship between them, but rather that the acquisition of these genes had occurred independently form the ancestor viruses. Since swine influenza surveillance was not practiced in the past in the region it is not known if SIVs of other subtypes have circulated in the country potentially contributing to the gene constellation of the described isolates, as it is not uncommon to have multiple subtypes of swine influenza viruses being present in the same region in the same year [16].

The rest of the genes apparently originated from different lineages of SIVs, best exemplified by the NA genes of three different sources (Figure 2).

Although the antigenic evolution of SIVs occurs at a lower rate than in case of human viruses [29,30], their frequent reassortation pose diagnostic and controlling problems. From the diagnostic point of view, the widely used haemagglutination inhibition tests can be affected by the degree of homology between the antigen used/investigated in a testing [9]. Recognizing the importance of the presence of H1N2 subtype viruses in the member countries there are trivalent vaccines already available in the EU for controlling SI, which comprise all three subtypes of SIV (H1N1, H3N2, and human-like H1N2; http://www.ema.europa.eu/pdfs/vet/opinion/66108409en.pdf; http://www.ema.europa.eu/pdfs/vet/opinion/66111609en.pdf). There is weak cross-reactivity between human-like and avian-like H1N2 SIVs induced immune responses [11]. Therefore, the antigenic composition of the vaccines needs regular re-evaluation and update based on the epidemiological data on swine influenza, taking into account the growing number of influenza virus variants circulating in pigs, including the new H1N1 viruses http://www.pighealth.com/influenza.htm#Outbreaks. The knowledge on the genetic constellation of SIVs contributes to the comprehension of these subjects and to the development of improved prevention strategies. Therefore, the molecular surveillance of SIVs is an important and rational task. Moreover, in order to comprehend the evolution and ecology of influenza viruses retrospective analyses are also indispensable, as exemplified by the numerous studies aiming at the understanding of the appearance of pandemic H1N1 influenza viruses [31].

A recent surveillance investigation for the occurrence of SIVs in five European countries demonstrated the presence of multiple subtypes in each country [11]. Furthermore, it showed that the so-called 'second-generation' (avian-like reassortant) H1N2 SIVs, that are alike the latest Swedish and recent Danish SIV isolates [13], were not common in the investigated pig herds. In fact, after their first notice in Europe in 1987 [7], they had not spread beyond their region of isolation [1]. Swine influenza outbreaks are quite rare in Sweden, thus, the number of isolates subjected to detailed genetic studies is limited. In order to improve the knowledge on the incidence of SIVs in the region, a working group/network was established with the participation of the Nordic countries (Denmark, Finland, Norway, and Sweden) in order to harmonize and facilitate research and diagnostic activities related to influenza in pigs by means of information exchange and joint activities.

## Competing interests

The authors declare that they have no competing interests.

## Authors' contributions

IK took part in conception, performed sequence analyses, alignments, phylogenies, drafted and wrote the manuscript. AB took part in conception, performed sequence analyses, alignments, phylogenies, contributed to and revised the manuscript. FW and EE obtained the viruses, provided core data, contributed to the interpretation of the findings and to the writing of the manuscript. GM carried out the PCR and sequencing reactions, and participated in data analysis. SB critically revised the manuscript and gave the final approval for publication. PW took part in conception, contributed to and revised the manuscript. All authors read and approved the final manuscript.
